# Inhibition of Glutamate Release, but Not of Glutamine Recycling to Glutamate, Is Involved in Delaying the Onset of Initial Lithium-Pilocarpine-Induced Seizures in Young Rats by a Non-Convulsive MSO Dose

**DOI:** 10.3390/ijms222011127

**Published:** 2021-10-15

**Authors:** Marek J. Pawlik, Blanca I. Aldana, Lautaro F. Belfiori-Carrasco, Marta Obara-Michlewska, Mariusz P. Popek, Anna Maria Czarnecka, Jan Albrecht

**Affiliations:** 1 Department of Neurotoxicology, Mossakowski Medical Research Institute, Polish Academy of Sciences, Pawińskiego 5, 02-106 Warsaw, Poland; mobara@imdik.pan.pl (M.O.-M.); mpopek@imdik.pan.pl (M.P.P.); aczarnecka@imdik.pan.pl (A.M.C.); 2 Department of Drug Design and Pharmacology, Faculty of Health and Medical Sciences, University of Copenhagen, Universitetsparken 2, 2100 Copenhagen, Denmark; blanca.aldana@sund.ku.dk (B.I.A.); vch443@ku.dk (L.F.B.-C.)

**Keywords:** epilepsy, glutamatergic transmission, glutamine synthesis, methionine sulfoximine, metabolomics

## Abstract

Initial seizures observed in young rats during the 60 min after administration of pilocarpine (Pilo) were delayed and attenuated by pretreatment with a non-convulsive dose of methionine sulfoximine (MSO). We hypothesized that the effect of MSO results from a) glutamine synthetase block-mediated inhibition of conversion of Glu/Gln precursors to neurotransmitter Glu, and/or from b) altered synaptic Glu release. Pilo was administered 60 min prior to sacrifice, MSO at 75 mg/kg, i.p., 2.5 h earlier. [1,2-^13^C]acetate and [U-^13^C]glucose were i.p.-injected either together with Pilo (short period) or 15 min before sacrifice (long period). Their conversion to Glu and Gln in the hippocampus and entorhinal cortex was followed using [^13^C] gas chromatography-mass spectrometry. Release of in vitro loaded Glu surrogate, [^3^H]d-Asp from ex vivo brain slices was monitored in continuously collected superfusates. [^3^H]d-Asp uptake was tested in freshly isolated brain slices. At no time point nor brain region did MSO modify incorporation of [^13^C] to Glu or Gln in Pilo-treated rats. MSO pretreatment decreased by ~37% high potassium-induced [^3^H]d-Asp release, but did not affect [^3^H]d-Asp uptake. The results indicate that MSO at a non-convulsive dose delays the initial Pilo-induced seizures by interfering with synaptic Glu-release but not with neurotransmitter Glu recycling.

## 1. Introduction

In the mammalian brain, glutamine synthetase (GS) is bimodaly involved in the regulation of excitatory, glutamatergic transmission [1,2]. On the one hand, GS-mediated conversion of Glu and ammonia to Gln in astrocytes neutralizes Glu newly released from neurons. On the other hand, a portion of Gln newly formed in astrocytes is transferred back to neurons by specific Gln transporters catalyzing its astrocytic release (system N) and neuronal uptake (system A), respectively [3,4,5]. Here, it enriches the neurotransmitter pool of Glu: the above sequence of events are defined as the glutamine–glutamate cycle (GGC).

Methionine sulfoximine (MSO) is an irreversible GS inhibitor [6]. Acute GS inhibition by high MSO doses rapidly stimulates glutamatergic transmission, inducing convulsions and seizures [6,7,8], while GS inactivation associated with prolonged MSO treatment is accompanied by hippocampal sclerosis and spontaneous recurrent seizures (SRS) resulting from inefficient Glu uptake, a status manifesting chronic temporal lobe epilepsy (TLE) [7,9,10,11]. GS loss in brain-vulnerable regions (mainly hippocampus) has also been found to correlate with advanced TLE induced by other chemical stimuli, including pilocarpine (Pilo) [12,13,14] or kainic acid (KA) [15].

On the other hand, exogenously added Gln will induce a long-lasting, glutamatergic transmission-bolstering effect. This has been directly demonstrated in striatal cortical slices [16], but has also been recorded in epilepsy models: in entorhinal cortical slices derived from Pilo-treated chronic epileptic rats [17], in rats with KA-induced seizures [18], and in an in vitro model of post-traumatic epilepsy [19]. The role of Gln in promoting excitatory transmission and seizures has also been demonstrated in experiments employing low MSO doses causing moderate inhibition of GS. Low MSO depressed glutamatergic transmission and epileptiform discharges in cerebral cortical slices in vitro [20,21]. In vivo, intracerebral administration of MSO reduced amygdala kindling-induced seizures [22].

Pilocarpine is considered a particularly valuable model for examining the development of temporal lobe epilepsy both in adult [23,24] and juvenile rats [25,26,27,28]. We recently have demonstrated that in 24-day rats treated with lithium-pilocarpine (Li-Pilo) following exactly the procedure described in [26], pretreatment with MSO at a low dose (75 mg/kg b.w.) which produced no convulsions, delayed the onset of and attenuated the initial convulsive and electrographic seizures [29]. It appeared reasonable to speculate that MSO at this low dose inhibited an aspect of glutamatergic transmission critical for provoking the initial seizures. In the present study, we test two not mutually exclusive hypotheses regarding the mechanism by which MSO could induce the inhibition. The first was that MSO interferes with the GGC in a way affecting de novo Glu synthesis in this model. To test this hypothesis, [^13^C] mass spectrometry was employed to follow incorporation in vivo of ^13^C labelled metabolic precursors: acetate and glucose, to Glu and Gln. This procedure has previously been used to study Glu synthesis and metabolism in models of brain pathologies associated with enhanced glutamatergic transmission [30,31,32,33,34,35,36], including treatments with convulsive MSO dose in vivo [30] and in vitro [36,37,38].

The second hypothesis was that MSO interferes with the availability of Glu at the synapse by modifying its release and/or reuptake. Here, we took into consideration earlier published data demonstrating that MSO affects Glu transport in various brain preparations in vivo [39] and in vitro [40,41]. To account for this hypothesis, we tested the effect of MSO on the uptake and release of the newly taken up ^3^H-labelled Glu surrogate, d-aspartate ([^3^H]d-Asp) in ex vivo brain slices derived from rats treated with a low dose of MSO. High, depolarizing potassium concentrations were used as a generic release stimulus.

## 2. Results

### 2.1. Behavioral Assessment of Seizures

**Figure 1:**
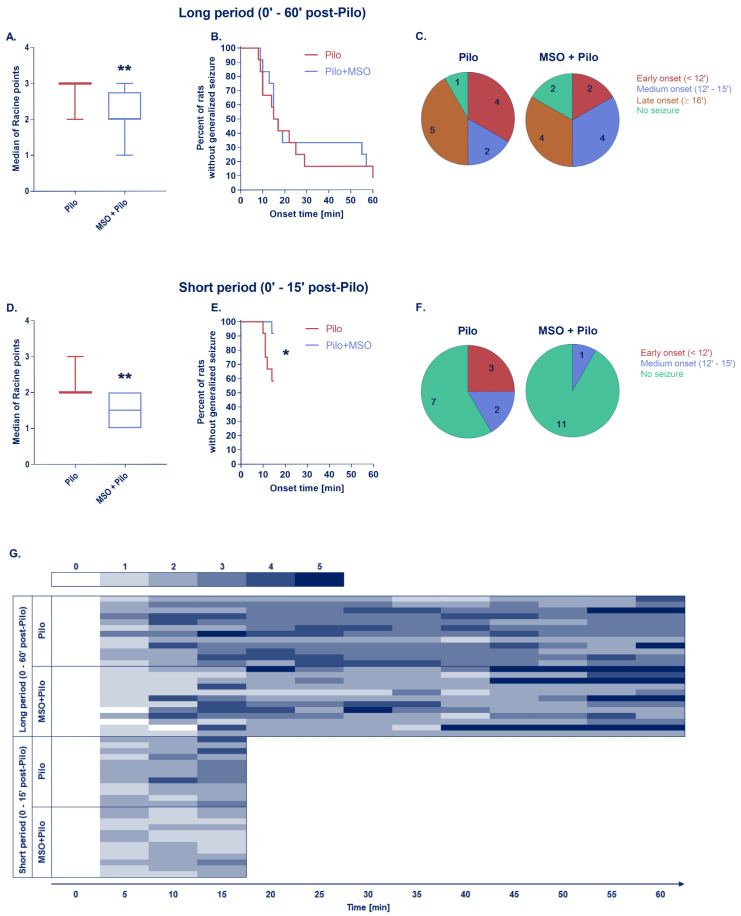
Box charts show the averaged magnitudes of seizures presented as a median of Racine points over a long period (**A**) or short period of observation (**D**), ** *p* < 0.01, Mann–Whitney test; survival plots show latency to the onset of the first generalized seizure among each group (**B**,**E**), * *p* < 0.05, Gehan–Breslow–Wilcoxon test; pie charts present the onset periods of the first generalized seizure—colors indicate distinct periods, numbers represent quantity of animals in each onset period (**C**,**F**); heat map shows detailed seizure dynamic for each animal in Racine scale (**G**); Pilo, MSO+Pilo N = 12 for both time periods.

MSO significantly reduced behavioral symptoms of seizures throughout the whole 60-min observation period (Figure 1A) as well as during the short 15-min period (Figure 1D), the magnitude similar to that observed previously [29]. MSO significantly delayed the onset of generalized seizure in the “short period” group (Figure 1E,F) and tended to delay the onset in the “long period” group (Figure 1B,C), again, in a manner similar to that previously described [29]. As shown in the heat map (Figure 1G), the severity of seizures between individuals varied substantially throughout whole observation period.

### 2.2. Generation of Glu and Gln from [U-^13^C]glucose or [1,2-^13^C]acetate

#### 2.2.1. Glutamate

**Figure 2:**
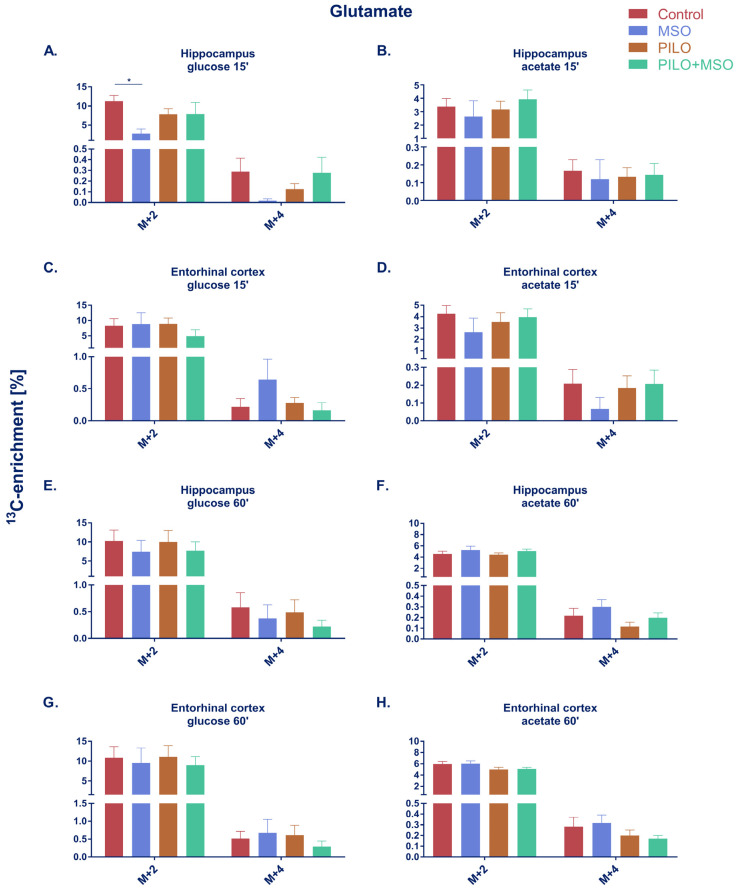
Percentage of ^13^C-enrichment in glutamate derived from [U-^13^C]glucose (**A**,**C**,**E**,**G**) or [1,2-^13^C]acetate (**B**,**D**,**F**,**H**) in hippocampus and entorhinal cortex of TLE model rats. * *p* < 0.05, one-way ANOVA with Tukey’s correction; Control, MSO, Pilo, MSO+Pilo N = 6; mean ± SEM. Designations “15” and “60” refer to the short (**A**–**D**) and long period (**E**–**H**) of Pilo action and correspond to 150–165 min or 195–210 min time of action of MSO, respectively, rendering in each case a 15 min presence of the isotope in the body.

At 15 min after Pilo administration, ^13^C-enrichment of Glu from labelled glucose was drastically decreased by MSO both after the first (M+2) and the second turnover (M+4) (Figure 2A). In the latter, the levels of 5 out of 6 samples were below the detection limit of gas chromatography coupled to mass spectrometry (GC-MS). However, Pilo treatment alone did not induce changes in the enrichment (Pilo group), MSO failed to decrease the ^13^C-enrichment from glucose in Pilo-treated rats (MSO+Pilo group). No changes after either combination of treatments were apparent in the entorhinal cortex, except for a tendency towards increase of M+4 labeling in Glu (Figure 2C). A slight tendency to decrease of Glu labelling from acetate by MSO became apparent in the entorhinal cortex (Figure 2D) but not in the hippocampus (Figure 2B). Again, MSO failed to decrease the ^13^C-enrichment from acetate, leaving M+2 and M+4 Glu levels unchanged relative to the control in both brain structures. No noteworthy changes in Glu ^13^C enrichment were observed at 60 min after Pilo administration (Figure 2E,H). In both time points and brain structures, and under either condition, ^13^C-enrichment in Glu derived from labeled glucose was around two times higher than that derived from acetate.

#### 2.2.2. Glutamine

**Figure 3:**
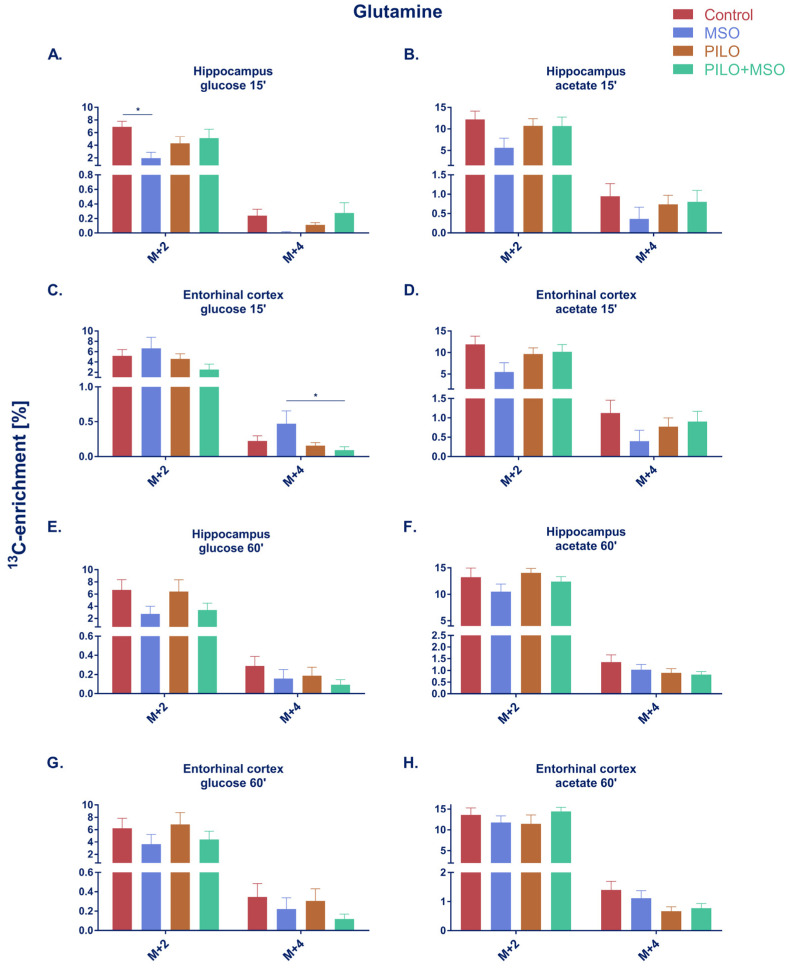
Percentage of ^13^C-enrichment in glutamine derived from [U-^13^C]glucose (**A**,**C**,**E**,**G**) or [1,2-^13^C]acetate (**B**,**D**,**F**,**H**) in the hippocampus and entorhinal cortex of TLE model rats. * *p* < 0.05, one-way ANOVA with Tukey’s correction; Control, MSO, Pilo, MSO+Pilo N = 6; mean ± SEM. Designations “15**′**” and “60**′**” refer to the short (**A**–**D**) and long period (**E**–**H**) of Pilo action and correspond to 150–165 min or 195–210 min time of action of MSO, respectively, rendering in each case a 15 min presence of the isotope in the body.

Similarly to glutamate, glutamine enrichment from ^13^C-labelled glucose in the hippocampus at 15 min was significantly reduced by MSO after the first turnover and almost indeterminable after the second turnover (4 out of 6 samples below detection limit) (Figure 3A). At 15 min, MSO failed to significantly affect glutamine enrichment from glucose in the entorhinal cortex (Figure 3C). A slight tendency towards decrease of Gln labelling by MSO was observed in both structures at 60 min when glucose was used as a precursor (Figure 3E,G). At 15 min, a tendency of MSO to decrease Gln labelling was also observed in acetate-derived metabolites in both structures; however, the effect did not reach statistical significance in either of the groups (Figure 3B,D). No change was observed in either structure after either treatment at 60 min (Figure 3F,H). Unlike Glu, where the majority of the newly labelled neurotransmitter originated from glucose, more than twice as much Gln was labelled from acetate than from glucose.

### 2.3. Glu and Gln Brain Tissue Content in MSO and/or Pilo-Treated Rats–A High Performance Liquid Chromatography (HPLC) Analysis

**Figure A1:**
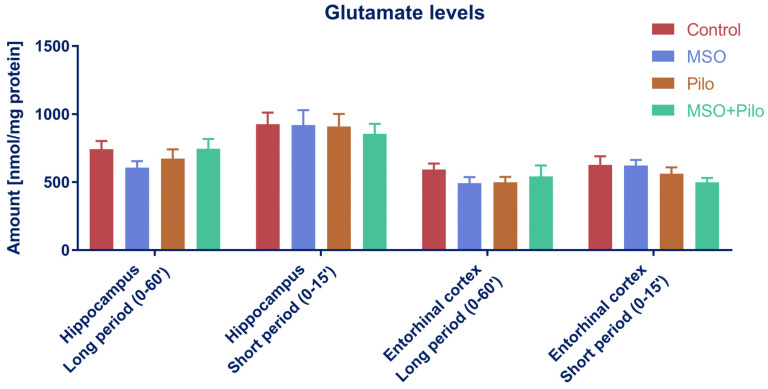
Glutamate levels in hippocampus and enthorhinal cortex of TLE model rats. One-way ANOVA with Tukey’s correction; Control, MSO, Pilo, MSO+Pilo N = 12; mean ± SEM.

In line with a previous report [42], none of the treatments produced statistically significant changes in hippocampal or entorhinal cortical Glu at either time point. Considering all the experimental variants, the Glu content ranged from 531.1 ± 89.4 in the entorhinal cortex to 788.1 ± 187.8 in the hippocampus (nmol/mg of protein, mean ± SD) (Figure A1).

### 2.4. [^3^H]d-Asp Efflux

**Figure 4:**
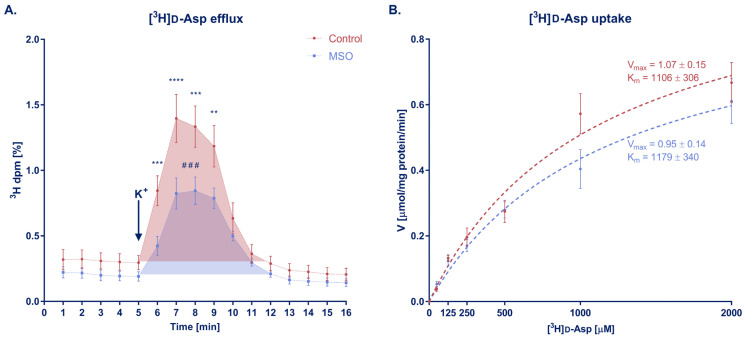
(**A**) [^3^H]d-Asp efflux from brain slices of rats that received MSO or vehicle (control group). Arrow indicates the moment when K^+^ depolarizing pulse was introduced. **, ***, **** *p* < 0.01, 0.001, 0.0001 respectively; #—statistical significance between areas under curve (AUC), one-tailed t-test; baseline for AUC comparison was set as a mean of fractions 1–5; baselines were compared using linear regression but changes between the slopes were not significant; control N = 5, MSO N = 6; mean ± SEM. (**B**) [^3^H]d-Asp uptake from brain slices of rats that received MSO or vehicle (control group). The dashed lines represent Michaelis–Menten kinetic curves; V_max_ and K_m_ constants were compared using t-test; control, MSO N = 7; mean ± SEM.

d-Asp release evoked by K^+^-induced depolarization was markedly lowered due to administration of MSO (Figure 4A). For four consecutive minutes after potassium pulse [^3^H]d-Asp levels in individual fractions were found to be significantly decreased in comparison with control. Area under the curve was on average 37% smaller in the MSO group as well (2.445 ± 0.139 vs. 3.888 ± 0.239 in control), indicating that the whole released pool of d-Asp was reduced. Baseline level of d-Asp was slightly decreased (0.204 ± 0.006% in MSO vs. 0.309 ± 0.005% in control) which may suggest that d-Asp transport is affected by MSO in normal conditions as well, however, these changes were statistically insignificant.

### 2.5. [^3^H]d-Asp Uptake

There was no change in the kinetics of [^3^H]d-Asp uptake, especially at lower concentrations of D-Asp (50–500 μM) (Figure 4B). At higher concentrations (1000–2000 μM), a slight decrease of the uptake rate was observed in the MSO-treated group; however, the impact of MSO was not pronounced enough to significantly change V_max_ or K_m_ parameters. This lack of effect of MSO confirms an earlier observation of unchanged Glu uptake in MSO-pretreated rat brain synaptosomes [40].

## 3. Discussion

Our primary hypothesis regarding the mechanism by which low dose MSO attenuates the initial seizures in the Li-Pilo model was that MSO decreases the synthesis of neurotransmitter Glu by reducing the formation of its precursor Gln. The hypothesis was verified by measuring the effects of MSO treatment on the incorporation in vivo of ^13^C labelled metabolic precursors: acetate and glucose to neurotransmitter Glu and Gln in the hippocampus and entorhinal cortex, the two structures involved in the propagation of pilocarpine-induced seizures [43]. The only statistically significant change observed throughout the study was a decrease of ^13^C -enrichment of both Glu and Gln from glucose in hippocampus in the period of 150–165 min after MSO administration alone. However, at the very same time point, MSO failed to modulate in either direction the enrichment from glucose in rats with Li-Pilo-evoked seizures. Otherwise, MSO did not induce any significant effect on ^13^C–enrichment from glucose nor acetate, in any animal group either at the short (15 min) or at the long period (60 min) of the development of initial seizures. It is intriguing that the decrease by MSO of ^13^C–enrichment from glucose at 15 min was not observed at the later time point. Tentatively, this may be due to the biphasic effect of MSO on GS activity, leaving open the time brackets of the system in the long period. A study with human GS revealed that initial competitive inhibition is a reversible process and is followed by irreversible activation [44]. The general absence of the effects of extrinsic factors became apparent notwithstanding the predictable metabolic fates of ^13^C glucose or ^13^C acetate in this experimental setting. Both preferential ^13^C–enrichment of Glu from glucose and of Gln from acetate are a good fit into the present views on the role of either of the compound as a metabolic precursor in neurons and astrocytes. Indeed, neurotransmitter pool of Glu is thought to be preferentially synthesized from glucose. The de novo synthesis may occur directly in neurons [45], or in astrocytes. The synthesis in astrocytes depends on anaplerosis, i.e., reactions providing a net increase in TCA cycle intermediates [46]. The primary anaplerotic enzyme in the brain, pyruvate carboxylase (PC), which serves as the main pathway of de novo glutamate synthesis is exclusively expressed in astrocytes [47,48]; reviewed by Schousboe et al. [49]. With regard to Gln, which is exclusively synthesized in astrocytes, its relatively high labelling from acetate supports the preferred [50], albeit not exclusive [36,38] astrocytic metabolism of acetate.

Most previous studies in which MSO was found to significantly affect generation of Glu from [^13^C] metabolic intermediates (glucose and/or acetate) have analyzed effects of direct application of MSO to in vitro preparations, at concentrations ranging from 1 to 10 mM [36,37,38]. Significant reduction of ^13^C glucose flux in the brain in vivo was reported upon 6 h after injection of 150 mg/kg MSO i.p. [30], which is a sub-acute dose reported to cause convulsion [51]. The here used 75 mg/kg i.p. dose of MSO administered for 165–210 min was comparatively low, likely below the threshold required to produce a significant effect. It is intriguing, however, that the reduction of ^13^C–enrichment of Gln and Glu from glucose at the 15 min time point by MSO found in control rats was not observed in Pilo-treated rats. The mechanism by which Pilo abrogated the effect of MSO could be related to deregulation of glucose metabolism. Depending upon the experimental setting, Pilo-induced seizures in their initial phase are accompanied either by an increased [52,53,54,55] or decreased cerebral glucose uptake/consumption [56,57,58]. Whatever their direction, the changes in glucose metabolism inflicted by Pilo-induced seizures may interfere with the effects of MSO. It is likely that in the MSO+Pilo group, the effect of MSO is additionally masked by Pilo-evoked release of a large pool of unlabeled Glu, indeed, a small tendency towards decrease of a ^13^C labelled Glu pool is noted at 15′ post-Pilo (Figure 2A).

The observation that MSO added at a low, non-convulsive dose did not significantly affect the ^13^C–enrichment of Gln or Glu in Li-Pilo-stimulated brain slices substantiated investigation of GGC-bypassing effects of MSO on Glu release and uptake. The key observation made was that MSO markedly reduced the release of newly loaded [^3^H]d-Asp from ex vivo brain slices subjected to the isotonic depolarization stimulus (75 mM K^+^). [^3^H]d-Asp release has been observed to mimic fairly well depolarization evoked release of the synaptic vesicular pool of Glu in brain slices [59] and cultured neurons [60]. Earlier, this paradigm proved useful in explaining variable responses of glutamatergic transmission in different models of hepatic encephalopathy [61]. The present results suggest that in the Li-Pilo-treated rats in situ, MSO attenuated the glutamatergic aspect of the initial seizures by directly inhibiting Glu release, albeit interactive interference of MSO and Pilo with the metabolism of Glu precursors to Glu described in the previous paragraph cannot be excluded. Since induction and propagation of seizures by Pilo is subject to a complex mechanism encompassing a yet not fully resolved interplay of cholinergic and glutamatergic stimulation [62,63], it is clear that the reduction of the release, noted in brain slices obtained from MSO-treated rats in response to a single depolarizing stimulus, can only be considered as a rough approximation of its seizure-attenuating effect of Pilo in situ. Of note, basal, unstimulated [^3^H]d-Asp release from the slices was not affected by MSO exposure (Figure 4), a finding contrasting with stimulation of Glu release observed in different experimental settings at pro-convulsive MSO doses [39,41]. The above discrepancy underscores the dose-dependent duality of the effects of MSO.

Clearly, caveats of the interpretation that potassium-stimulated [^3^H]d-Asp release genuinely reflects seizure-induced synaptic release of endogenous Glu remain to be addressed. If the interpretation is correct and if MSO-evoked reduction of synaptic Glu release is sustained throughout development of TLE to its full-blown stage, MSO would be a valuable addition to the hotly debated list of antiepileptic drugs with antiepileptogenic activity [64,65]. The fact that MSO is relatively much less neurotoxic in primates than in rodents [8] substantiates further search into its antiepileptogenic potential. Clearly, studies towards this translational goal will have to encompass measurements of the effects of repeated reinjections of MSO in the time period following induction of the initial seizures.

The mechanism by which MSO reduces [^3^H]d-Asp (Glu) release deserves separate studies. Synaptic Glu release is a complex phenomenon consisting of an array of tightly controlled steps including Glu transport, synaptic vesicle formation, fusion, neurotransmitter release and vesicle recycling via endocytosis at the active zone (for recent reviews see [66,67,68]); either of the above mentioned aspects deserves careful consideration.

It cannot be excluded that modulation by MSO of glutamatergic transmission involves Gln synthesis-bypassing mechanisms other than synaptic Glu release as well. MSO inhibits not only GS, but also γ-glutamylcysteine synthetase, an enzyme diverting a significant portion of Glu towards glutathione synthesis [69]. MSO was also found to variably affect Gln fluxes in cultured astrocytes [70], and brain cortex slices [71]. However, the abovementioned findings were obtained in experimental conditions not easily translatable to those recorded in ex vivo slices in the present study.

Collectively, notwithstanding a number of caveats to be resolved, the results of the present study may be best interpreted as indicating that MSO at a non-convulsive dose delays, and possibly attenuates, the initial Pilo-induced seizures by interfering with mechanism of Glu release. By contrast, the results do not support the view that the “canonical” glutamine synthetase-inhibiting activity of MSO plays an essential role in the attenuation of the seizures at this stage.

## 4. Materials and Methods

### 4.1. Juvenile Rat Li^+^-pilocarpine TLE Model

The procedure was essentially as described previously [26,29] with minor modifications. At postnatal day 23 (P23), male Sprague Dawley rats (the animal colony of the Mossakowski Medical Research Institute, Polish Academy of Sciences in Warsaw) were injected intraperitoneally (i.p.) with lithium carbonate (222 mg/kg; Sigma-Aldrich, Steinheim, Germany) dissolved in saline (pH equalized to 7.4). At P24, 18–20 h after Li+ treatment, animals were injected i.p. with methyl-scopolamine (1 mg/kg; Sigma-Aldrich, Steinheim, Germany) and thirty minutes later with pilocarpine (40 mg/kg; Sigma-Aldrich, Steinheim, Germany). From then on, the animal’s behavior was continuously monitored utilizing five-stage Racine scale [72]: 1: mouth and facial movement, 2: head nodding, 3: forelimb clonus, 4: rearing with forelimb clonus, 5: rearing and falling with forelimb clonus, considering stages 1–3 as a focal and 4–5 as a generalized seizure. Rats were decapitated either 15′ (short period) or 60′ (long period) after Pilo administration (see also Section 4.4). Control rats that did not receive Pilo were given equal volumes of saline at the same time.

### 4.2. Pretreatment with MSO

MSO (Sigma-Aldrich, Steinheim, Germany) was dissolved in saline and administered i.p. at 75 mg/kg. Rats that were used in experiments with Pilo received MSO 150 min before Pilo; thus, 165 min or 210 min before decapitation. Rats that were used in experiments with Pilo received MSO 150 min before Pilo; thus, 165 min (Figure 2 and Figure 3A–D) or 210 min (Figure 2 and Figure 3E–H) before decapitation. Rats used for ex vivo experiments with brain slices received MSO 150 min before decapitation (Figure 4). Control rats that did not receive MSO were given equal volumes of saline at the same time.

### 4.3. Metabolic Studies In Vivo

**Scheme 1:**
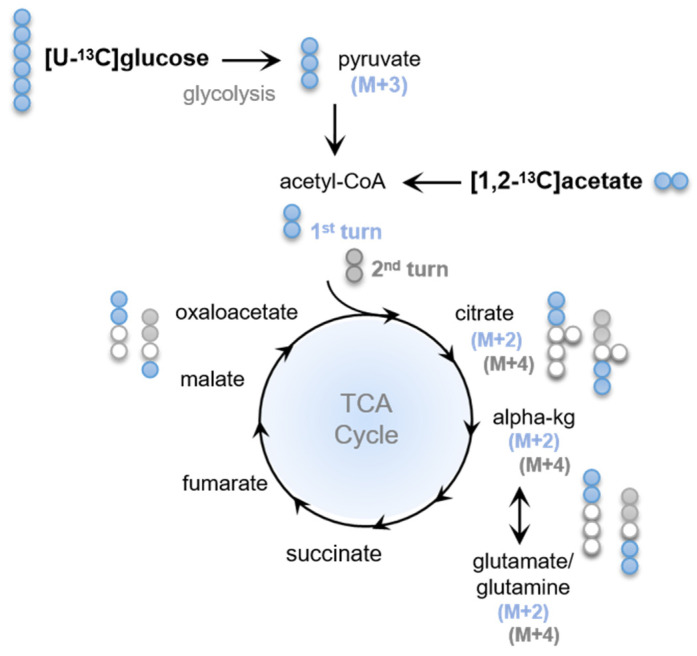
Schematic representation of the main labelling patterns obtained after incubation with [U-^13^C]glucose or [1,2 ^13^C]acetate. [U-^13^C]glucose is taken up by cells in the brain tissue and metabolized to [U-^13^C]pyruvate (M+3) during glycolysis. [U-^13^C]pyruvate which in turn can be metabolized in the tricarboxylic acid (TCA) cycle, entering as [1,2-^13^C]Acetyl-Coenzyme A ([1,2-^13^C]Ac-CoA) which reacts with unlabelled oxaloacetate and, hence, double-labelled (M+2) TCA cycle intermediates are produced, illustrated as blue circles. [1,2-^13^C]acetate can also enter the TCA cycle via [1,2-^13^C]Ac-CoA and generate double-labelled intermediates. The labelled neuroactive amino acid [4,5-^13^C]glutamate (M+2) can be formed from α[4,5-^13^C]ketoglutarate (α-kg M+2). From glutamate (M+2), [4,5-^13^C]glutamine (M+2) can be synthesized in the astrocytes by glutamine synthase (GS). In the second turn of the TCA cycle, [1,2-^13^C]Ac-CoA reacts with oxaloacetate M+2 formed in the first turn of the cycle, resulting in the production of M+3 and M+4 labelled metabolites (depicted with grey circles).

The following procedures were based on previously published papers [33,73]. The main labelling patterns of the metabolites from [U-^13^C]glucose or [1,2-^13^C]acetate in the experimental setting of the present investigation are detailed in Scheme 1.

Rats divided into four groups (control, MSO, Pilo, MSO+Pilo) received a single i.p. dose of [U-^13^C]glucose (543 mg/kg) or [1,2-^13^C]acetate (504 mg/kg) (Cambridge Isotope Laboratories, Tewksbury, MA, USA) dissolved in saline, either at the same time as Pilo (short period) or 45 min after the injection of Pilo (long period). All animals were decapitated after 15 min. Therefore, the precursors were present in the body at the short and in the long period of Pilo action. Brain tissue was immediately dissected and frozen in liquid nitrogen. Frozen hippocampi and entorhinal cortex samples were homogenized in 2 mL of ice-cold 70% ethanol and then centrifuged for 20 min at 20,000× *g*. Supernatant was collected, lyophilized, and used for amino acids analysis in GC-MS and HPLC; pellets were used for protein determination with the BCA assay (Thermo Fisher Scientific, Waltham, MA, USA).

#### 4.3.1. Metabolic Mapping Using GC-MS

Lyophilized extracts from hippocampus and entorhinal cortex tissue were resuspended, acidified to pH 1–2 with HCl and subsequently evaporated to dryness using nitrogen. With the use of 96% ethanol and benzene, the analytes were extracted, evaporated again to dryness and derivatized with 14% DMF/86% MTBSTFA [74]. Gas chromatography (GC, Agilent Technologies 7820A chromatograph, J&W GC column HP-5MS, parts no. 19091S-433, Santa Clara, CA, USA) coupled to mass spectrometry (MS, Agilent Technologies, 5977E, Santa Clara, CA, USA) was used to separate and analyze the samples. With the use of unlabeled standards, metabolites of interest were corrected to their ^13^C natural abundance in order to assess their isotopic enrichment. Calculations were performed according to [75]. Data integration and enrichment determination was performed with the software MassHunter Quantitative Analysis software v.6.0.3881 (Agilent Technologies, Santa Clara, CA, USA). Data are presented as labelling (%) of M+ X, where M is the mass of the unlabelled molecule and X is the number of labelled C-atoms in a given metabolite.

#### 4.3.2. Quantitative Determination of Intracellular Amino Acids by HPLC

Tissue extracts, previously resuspended in water, were separated by reverse-phase HPLC using an Agilent ZORBAX Eclipse plus C18 column (4.6 × 150 mm, particle size 3.5 μm; 959,963–902, Agilent Technologies, Santa Clara, CA, USA) in an Agilent 1260 Infinity system coupled to a 1260 Infinity fluorescence detector (Agilent Technologies, Santa Clara, CA, USA) as described previously [76]. Briefly, samples were derivatized with o-phthaldialdehyde and separation was performed with a mobile phase gradient consisting of a mixture of buffer A (10 mM Na_2_HPO_4_: 10 mM Na_2_B_4_O_7_, pH 6.9; 5 mM NaN_3_) and buffer B (acetonitrile 45%: methanol 45%: water 10%, v:v:v). Amino acids quantification was performed using standards containing a mixture of the amino acids of interest on increasing concentrations. The acquired data was normalized to the protein concentration in the dry tissue samples. Effects of MSO on the Gln content could not be accurately assessed because the position of the MSO peak which was in much excess over Gln largely overlapped with the position of the latter.

### 4.4. [^3^H]d-Asp Studies Ex Vivo on Acute Brain Slices

Rats anesthetized with isoflurane (Baxter, Deerfield, IL, USA) were decapitated, the brain was immediately removed, and cortices were cut into 350 μm thick slices, using McIlwain tissue chopper. The slices were pre-incubated for 30 min in the Krebs buffer (37 °C, aerated with 95% O_2_ and 5%CO_2_, pH 7.4), composed of [mM]: 118 NaCl, 25 NaHCO_3_, 4.7 KCl, 1.2 KH_2_PO_4_, 2.5 CaCl_2_, 1.2 MgSO_4_, 10 glucose. After preincubation, slices were used either for uptake or efflux measurement protocols, employing the radioactive [^3^H]d-Aspartate ([^3^H]d-Asp) as the non-metabolised analogue of glutamate.

#### 4.4.1. [^3^H]d-Asp Efflux Assay

The [^3^H]d-Asp efflux was assayed based on the method previously described [77], with modifications. [^3^H]d-Asp efflux was measured after 15 min incubation in Krebs buffer containing 1.4 µCi/mL [^3^H]d-Asp (Perkin-Elmer, Waltham, MA, USA) and unlabeled d-Asp (100 µmol/L). The slices were moved to a chamber perfusion system (Brandel, Gaithersburg, MD, USA) and rinsed with Krebs buffer at 0.5 mL/min rate. The initial fraction was collected for 20 min to establish baseline efflux. Perfusate samples were then collected for 20 min, at 1 min intervals. At the time period indicated in Figure 4A. (fractions 5 and 6), a depolarizing pulse was introduced by raising KCl concentration in the Krebs buffer to 75 mM, with simultaneous reduction of NaCl to 47.7 mM). Radioactivity contained in the preparation and released from brain slices were measured by a Wallac 1409 Liquid Scintillation Counter (Perkin-Elmer, Waltham, MA, USA).

#### 4.4.2. [^3^H]d-Asp Uptake Assay

The [^3^H]d-Asp uptake was assayed as previously described [78]. Briefly, the uptake was initiated by adding [^3^H]d-Asp, 0.1 μCi/1 mL (Perkin-Elmer, Waltham, MA, USA), to varying extracellular concentrations of unlabelled d-Asp (50–2000 μM). The incubation with the radioisotope was continued for 3 min and was terminated by rapid vacuum filtration through nitrocellulose filter disks (Millipore, Billerica, MA, USA), followed by flushing four times with 2 mL of ice-cold Krebs buffer. The slices were weighed, immersed in 4 mL of InstaGel scintillation fluid (Perkin-Elmer, Waltham, MA, USA), and the radioactivity of slices on filter disks was measured in the Wallac 1409 Liquid Scintillation Counter (Perkin-Elmer, Waltham, MA, USA).

**Scheme 2:**
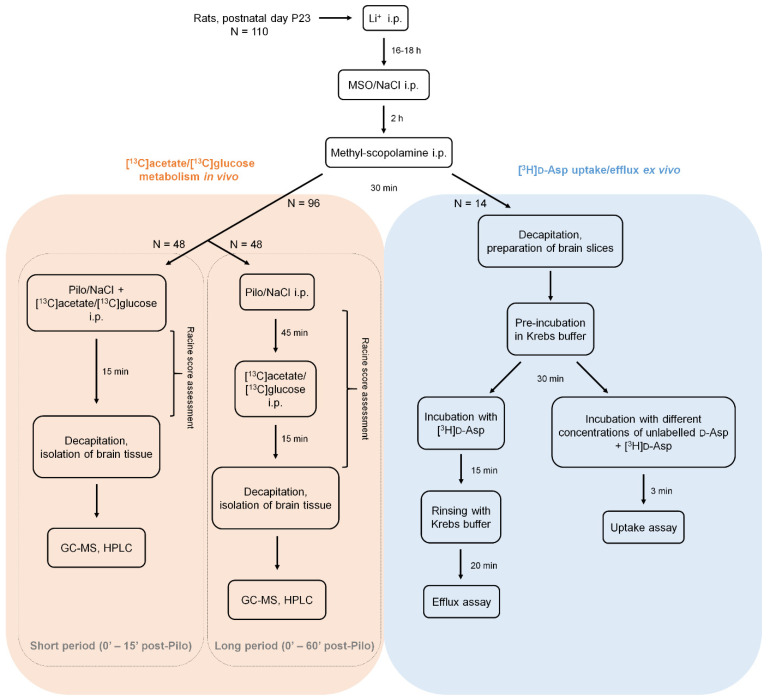
A diagram of the in vivo and ex vivo experiments in the Li-Pilo model. Male Sprague Dawley rats were administered sequentially with lithium, MSO or saline and methyl-scopolamine, starting at postnatal day 23 (P23). Afterwards, animals intended for metabolic experiments (left, orange panel) received a dose of a convulsive agent–pilocarpine or saline and were observed for 15 (short period groups) or 60 min (long period groups) and then decapitated. All rats in in vivo experiments were also injected with ^13^C-labelled metabolic precursor (acetate or glucose), 15 min prior to decapitation. Dissected brain tissue was homogenized and analyzed with gas chromatography coupled to mass spectrometry (GC-MS) and high performance liquid chromatography (HPLC). Animals intended for ex vivo experiments with ^3^H-labelled Glu surrogate, d-aspartate ([^3^H]d-Asp) (right, blue panel) were decapitated 30 min after methyl-scopolamine injection, the brain was isolated and immediately cut into slices. Acute brain slices were pre-incubated in Krebs buffer for 30 min, then incubated with [^3^H]d-Asp and subjected to efflux or uptake assays. In the metabolic studies (orange panel), a total of 96 animals were used: 6 animals in each group (Control, MSO, Pilo, MSO+Pilo): 6 × 4 groups × 2 ^13^C-precursors × 2 settings (long and short period). A total of 14 animals were used in the uptake/efflux experiments (blue panel).

A detailed diagram of experiments conducted in this study is presented in Scheme 2.

### 4.5. Data Evaluation

All statistical analysis were performed with Prism 7.0 (Graphpad Software Inc., La Jolla, CA, USA) software. T-tests or Mann–Whitney tests were used for comparisons of two groups (respectively for parametric and non-parametric data) and one-way ANOVA for more than two groups (all the data sets considered were parametric); the Gehan–Breslow–Wilcoxon test was used for comparison of survival curves. Multiple comparisons were followed by Holm–Šidák’s (multiple *t*-tests) or Tukey’s (ANOVA) post hoc tests. The significance level was set at *p* < 0.05. Heat map was prepared with Excel 2016 (Microsoft, Redmond, WA, USA).

## 5. Conclusions

The major novel observation of the present study is that MSO at a non-convulsive dose delays the initial Pilo-induced in a mode not discernably related to its canonical, glutamine synthetase-inhibiting activity. This observation is unique in that, as mentioned in the Discussion, previous therapeutic interventions with MSO in other diseases were based on its canonical mechanism of action. The present results strongly indicate that the seizure-ameliorating effect of MSO is due to its interference with Glu release.

## Data Availability

Data available on request.
